# Non-intubated Thoracoscopic Surgery—Pros and Cons

**DOI:** 10.3389/fsurg.2021.801718

**Published:** 2021-12-06

**Authors:** Miroslav Janík, Peter Juhos, Martin Lučenič, Katarína Tarabová

**Affiliations:** ^1^1st Department of Thoracic Surgery, University Hospital Bratislava and Slovak Medical University, Bratislava, Slovakia; ^2^1st Department of Anaesthesiology and Intensive Care Medicine, University Hospital Bratislava and Faculty of Medicine, Comenius University Bratislava, Bratislava, Slovakia

**Keywords:** Miroslav Janik, non-intubated VATS, ERAS, NITS, vats lobectomy

## Abstract

Pulmonary resection by video-assisted thoracoscopic surgery with single-lung ventilation has become a standardized modality over the last decades. With the aim to reduce surgical stress during operation procedures, some have adopted a uniportal approach in pulmonary resection as an alternative to multiportal VATS. The ERAS program has been widely spread to achieve even better outcomes. In 2004, Pompeo reported the resection of pulmonary modules by conventional VATS under intravenous anesthesia without endotracheal intubation. Within less than a decade thereafter, complete VATS pulmonary resections under anesthesia without endotracheal intubation had been reported for a range of thoracoscopic procedures. Avoiding tracheal intubation under general anesthesia can reduce the incidence of complications such as intubation-related airway trauma, residual neuromuscular blockade, ventilation-induced lung injury, impaired cardiac performance, and postoperative nausea. Numerous studies can be found especially from Asian countries, focusing on comparison of intubated and non-intubated procedures showing that non-intubated VATS could reduce the rate of postoperative complications, shorten hospital stay and decrease the perioperative mortality rate, indicating that non-intubated VATS is a safe, effective and feasible technique for thoracic disease. However, if we look closely at all studies, it is obvious that there are no significant differences between intubated and non-intubated surgery in terms of the standard procedures and maneuvers. In non-intubated procedures it can be less comfortable for the surgeon to manipulate in the thoracic cavity, but the procedural steps remain the same. All the differences between the intubated and non-intubated operation procedure are found in perioperative management of the patient. The patient is still in deep anesthesia during the procedure and hypecapnia can occur. It is easier to manage this if the patient is intubated. In addition, if a complication occurs during the operation and intubation is required, this can cause an emergent situation, which means that not all patients are suitable for such a procedure, especially those with severe emphysema, obese patients and those with a problematic oropharyngeal configuration-Mallampati score. Moreover, studies on non-intubated thoracic surgery point to shortened hospitalization, faster recovery etc. But there are also studies on intubated uniportal VATS procedures in combination with ERAS protocol showing shortened hospitalization and better outcome for patients. Currently, especially with the use of optical intubation canylas, totally intravenous anesthesia (TIVA), BIS and relaxometer, anesthesia is safe for avoiding airway injury, hypercapnia, and there is minimal risk of residual curarization as well as one of the postoperative lung complications such as microaspiration and atelectasis. In addition, the patient recovers rapidly from anesthesia and can be verticalised and mobilized a couple of hours after the operation. It is desirable to take into consideration what type of patient and what lung disease is suitable for non-intubated technique and what is more convenient for intubation.

## Introduction

Videoassisted thoracic surgical procedures have been developed during the past decades with the aim of reducing surgical stress on patients, improving patient outcomes in terms of radicality, but also in recovery. A standardized operating procedure has been established in the surgical treatment of benign and malignant lung diseases. Thoracoscopy is also considered to be a valuable staging and diagnostic procedure with minimal impact on the patient's funcionality. Minimally invasive approaches in thoracic surgery have undergone an evolution. To reduce the number of the ports entered and with the goal to minimize surgical stress, the uniportal VATS approach was introduced by Gonzales et al. (1).

Since Pompeo et al. published their first report on nodules resection by VATS without endotracheal intubation, and thereafter, complete VATS pulmonary resections under anesthesia without endotracheal intubation had been reported for a range of thoracoscopic procedures (2–6). According to some papers, intubated general anesthesia with one lung ventilation has recognizable complications including: postoperative sore throat, irritating cough, nausea and vomiting, ventilator-related complications, and impaired lung performance. To avoid the negative side effects of tracheal intubation and general anesthesia, non-intubated VATS techniques began to be considered as an alternative to general anesthesia with intubation (4, 7, 8).

To date, there are several reports on this method showing encouraging results in low morbidity, short hospital stay and no procedure-related mortality.

## Pros and Cons

### Advantages of NITS With Criticism

The rapid development of mini-invasive surgical approaches in thoracic surgery has brought several modalities in techniques used in various surgical procedures. Minimally invasive approaches have become standard in treatment. To improve patient outcomes, there is a need to minimize even these current mini-invasive modalities. Evolution in this field is respresented by non-intubated procedures, expecting further improvement in patient outcomes. It is possible to find several studies on this topic. These studies compare non-intubated procedures with intubated ones. Almost all of them compare common complications of thoracic procedure. The vast majority of them showed better results in hospital stay or mortality (9, 10). But it is obvious that the operation procedure is the same, no matter whether the patient is under general anesthesia with intubation or awake, or under anesthesia and not intubated. Surgical procedure depends on the customary practice of the surgeon and their skills, from a four or three port approach to uniportal most recently. The technique used does not differ, nor do tools and equipment.

The only notable difference and possible expectance in improvement of patient outcomes between intubated and non-intubated procedure is in anesthesiological management of the patient. Some papers emphasize the advantages of a non-intubated approach, assuming that it is possible to avoid complications associated with general anesthesia and intubation, such as tracheal injury, vocal cord injury, lung impairment due to artificial ventilation, intubation-associated discomfort, or risk of alveolar barotrauma. General anesthesia puts the patient at risk of residual neuromuscular blockade and postoperative ventilator dependency. The absence of general anesthesia may explain faster postoperative recovery, lower complication rates, and reduction of the stress hormones (11, 12). Several anesthesiological techniques for pain, sedation and local anesthesia management are well-described (13).

On the other hand, a large number of studies have proved fast recovery after mini-invasive thoracic surgery in patients who underwent a procedure under general anesthesia with intubation, especially when ERAS protocol was used. Comparing results, it is obvious that there is no significant difference in patient outcomes in terms of morbidity, surgery-related complications and hospital stay. Modern anesthesia using an optical endobronchial double lumen tube with the camera connected to a monitor during the intubation significantly reduces airway and vocal cord injury. Total intravenous anesthesia protocol with bispectral index monitoring provides reduction of anesthetic use and end-tidal anesthetic concentration, which leads to faster recovery from anesthesia (14, 15).

There is a risk of the postoperative need for artifitial ventilation in patients with severe COPD, if general anesthesia is used. In this group of patients, a non-intubated approach can be the option if the risk-benefit balance is in favor of surgery to turn the acute pathological condition.

### Disadvantages of NITS With Criticism

A non-intubated approach has some effects on respiration during surgery. The patient is deeply sedated but spontaneously ventilated. For non-intubated VATS it is inevitable to create iatrogenic pneumothorax to achieve adequate access and visualization. It necessarily leads to hypoventilation and decrease of pulmonary perfusion. This condition also causes a decrease of functional residual capacity and hypercapnia (16). Thus, some anesthetic techniques have been developed to mitigate these adverse effects (17).

The operation technique remains the same. A high complication rate related to the surgical procedure is not expected. VATS procedures, especially major pulmonary resections, are safe and effective surgical procedures. Despite the fact that these techniques are characterized by small incisions, it could cause major and potentially fatal complications if not promptly managed (18). The most serious and urgent situation requiring prompt action is massive bleeding from the pulmonary artery. The vast majority of bleeding is controllable by compression and if needed, there is still enough time to convert to thoracotomy. But occasionally, bleeding is uncontrollable and immediate thoracotomy with clamping of the PA is necessery. In such cases the anesthesiologist must be capable of intubating the patient in the lateral decubitus position. It requires skill and experience in this field, much like ideal anatomical conditions for intubation (Mallampati score).

Some limitations for non-intubated procedures may appear, such as extreme movements of the diaphragm and mediastinum in some patients, coughing, severe emphysema, or obesity. There is no absolute contraindication to general anesthesia. As non-intubated techniques require more experience, preparation and vigilance in thoracic surgery, patients must be carefully selected as candidates. It seems that for a non-intubated approach, ASA 2 patients are the most suitable. The Mallampati score in these patients should not exceed grade II. Patients with a body mass index of more then 30 are excluded. On the other hand, some papers also described thoracoscopic procedures using epidural anesthesia or local anesthesia in high-risk patients contraindicated for general anesthesia (19, 20).

## Discussion

Recent reports and studies have suggested that many surgical thoracic procedures are feasibile using non-intubated anesthetic techniques. Approval for these procedures is being sought as an alternative to the standard procedures performed under general anesthesia with intubation. The expected advantages should be the avoidance of airway and vocal cord injury, artifitial ventilation-related trauma, and minimizing the risk of postoperative complications. It is also expected that with no general anesthesia, which can affect the cellular and humoral immune response, it is possible to avoid the incidence of postoperative infections and it can also decrease the chance of tumor progression (21, 22). It is also claimed that recovery after non-intubated procedure is faster with fewer complication rates. Non-intubated procedures aim to minimize both surgery and anesthesia. Still, there are contraindications for this procedure such as morbid obesity, a Mallampati score of more than II or other anatomical deviations and pathologies, severe emphysema, pleural adhesions, extreme movements of diaphragm and mediastinum, or non-compliant patient. Even if a non-intubated approach can be an attractive alternative for some patients requiring thoracic surgery, some concerns arise. Even if the procedure is performed perfectly, crisis can occur, especially massive bleeding, which can lead to a disastrous outcome. After serious consideration, it is obvious that patients should be selected carefully since it is not suitable for all patients. Indications for such an approach are still unclear. In the era of modern anesthesia with adequate equipment, adverse effects are reduced, and using ERAS protocol, patient recovery is fast even after thoracic procedures using intubated general anesthesia.

It is still early to draw conclusions on the role of non-intubated techniques in thoracic surgery. Several studies showed encouraging results, but on the other hand, one must also consider the safety of actual general anesthesia using TIVA (totally intravenous anesthesia) and BIS monitoring and intubation techniques with an optical double lumen intubation tube.

## Conclusion

It seems that non-intubated procedures could be an alternative for the high-risk group of patients contraindicated for general anesthesia or impaired lung function with risk of ventilator dependence.

No doubt, thoracic procedures in a non-intubated way must be performed in a center with high experience and with an experienced staff. Although the incidence of conversion to intubated general anesthesia is low, a conversion protocol should be prepared in advance.

We can conclude that most of the thoracic surgical procedures will be performed under general intubated anesthesia as a standard, and the non-intubated approach remains as an alternative for selected patients.

## Author Contributions

All authors listed have made a substantial, direct, and intellectual contribution to the work and approved it for publication.

## Conflict of Interest

The authors declare that the research was conducted in the absence of any commercial or financial relationships that could be construed as a potential conflict of interest.

## Publisher's Note

All claims expressed in this article are solely those of the authors and do not necessarily represent those of their affiliated organizations, or those of the publisher, the editors and the reviewers. Any product that may be evaluated in this article, or claim that may be made by its manufacturer, is not guaranteed or endorsed by the publisher.
